# Cooling e-cigarette flavors and the association with e-cigarette use among a sample of high school students

**DOI:** 10.1371/journal.pone.0256844

**Published:** 2021-09-01

**Authors:** Danielle R. Davis, Meghan E. Morean, Krysten W. Bold, Deepa Camenga, Grace Kong, Asti Jackson, Patricia Simon, Suchitra Krishnan-Sarin

**Affiliations:** 1 Department of Psychiatry, Yale School of Medicine, New Haven, CT, United States of America; 2 Department of Emergency Medicine, Yale School of Medicine, New Haven, CT, United States of America; University of Wisconsin-Madison, UNITED STATES

## Abstract

**Introduction:**

E-liquid flavor is typically presented by flavor category (e.g. menthol, mint, fruit, dessert). Cooling sensations produced by flavor additives such as menthol enhance appeal of e-cigarettes among youth, but not all e-liquids that produce cooling sensations are labeled as menthol. Sensory experiences produced by flavors may allow for a new way to capture e-cigarette flavor use. This study aims to examine use of flavors that produce cooling sensations among youth and its association with e-cigarette use behaviors.

**Methods:**

A 2019 survey of high school students (n = 4875) examined use of e-cigarette flavors that produced cooling sensations (cooling flavors) among past 30-day e-cigarette users. E-cigarette use behaviors (flavor use, nicotine use, frequency of use) were examined between those who did and did not use cooling flavors. A binary logistic regression was used to examine associations between vaping frequency, nicotine (vs. non-nicotine) use, and vaping cooling flavors while controlling for demographics, number of flavors vaped in the past month, and vaping age of onset.

**Results:**

51.6% (n = 473/916) of the analytic sample endorsed vaping cooling flavors. There were no demographic differences by vaping cooling flavors. Vaping cooling flavors was associated with vaping more frequently (AOR:1.04,95% CI:1.03,1.05) and vaping nicotine (AOR:2.37,95% CI:1.53,3.67).

**Conclusion:**

Vaping cooling flavors was associated with greater nicotine vaping and frequency of e-cigarette use. Assessing sensory experience, such as cooling, in addition to flavor category may more fully capture e-cigarette flavor use and its impacts on youth e-cigarette use behaviors.

## Introduction

E-cigarettes are the most commonly used tobacco product by youth [1]; 19.6% of high school students reported past-month use in 2020 [2]. Importantly, flavors are a well-documented contributor to e-cigarette use and appeal among youth [3]. Recent evidence suggests that the most widely used flavors by high school students is fruit (73.1%), followed by mint flavors (55.8%), menthol flavors (37.0%), and dessert/candy/sweets (36.4%) [2]. Evidence suggests that flavor additives alter the sensory experience of tobacco products and make them more palatable [4–6].

E-cigarette flavors often are classified by broad categories (e.g. fruit, dessert, menthol/mint, tobacco) [7] and e-cigarette use patterns are examined by these flavor categories. However, flavor is complex and can be broken down into multiple components: taste, smell, and chemesthesis (the chemical sensitivity of a flavor on skin and mucous membranes; i.e. thermal perception, pain, touch perception) [8]. Thus, broad flavor categories that emphasize the name of the flavor (e.g., fruit) may not well capture all components of flavor. For example, some flavors commonly marketed by categories such as fruit or candy (e.g., strawberry chill) may also contain additives that produce specific sensory effects that influence the flavor experienced.

Menthol is a flavor additive that produces cooling and analgesic sensations in the mouth and throat and has been shown to increase the appeal of tobacco product. In the past, tobacco companies have advertised products containing menthol to youth and young adults because menthol’s cooling and analgesic sensory characteristics have been shown to facilitate smoking initiation and nicotine intake [9]. Menthol additives in e-liquids reduce the aversiveness, harshness, and irritation associated with vaping nicotine, as well as increase liking of e-cigarettes in both youth and adults [5, 6, 10]. However, many e-cigarettes that are not labeled as menthol flavored often use descriptors such as ice, cool, chill, and freeze, implying that the product produces cooling sensations. Further, menthol-like cooling sensations can also be produced by other flavoring chemicals like menthone, eucalyptol, peppermint oil, and odorless compounds such as WS-3, all of which have been detected in various tobacco products [11, 12]. Many tobacco products, including e-cigarettes, that are not labeled as menthol flavored have been shown to contain low levels of menthol and/or other cooling additives [13] and even low levels of these cooling additives, which at these low levels may not have been classified as a characterizing flavor, have been shown to increase e-cigarette appeal [5, 14].

If sensory experiences to flavors drives the appeal of e-cigarettes and other tobacco products in addition to the definition of a “characterizing flavor” currently used by the Food & Drug Administration (FDA) and the European Union (EU), then perhaps flavors should be categorized based on sensory experiences in addition to characterizing flavors and currently used discrete flavor categories. Given that the FDA is considering a menthol ban in combustible tobacco products [15], it is important to consider any constituents that produce a similar *cooling sensory experience* to menthol (e.g. eucalyptol, WS-3) in such regulations. Otherwise, constituents that may provide a similar cooling sensory experience to menthol, but not necessarily the characterizing flavor, could still be used to increase product appeal. No study has yet evaluated if the sensory experiences can be used to examine use of flavors instead of or in addition to flavor categories (e.g. menthol/mint, fruit, candy). This may be especially important to evaluate among youth to whom tobacco companies have targeted flavored products in the past.

The aim of the current study is to (1) identify among e-cigarette using youth percentage that use e-liquids that produce cooling effects (i.e. cooling flavors) by querying specifically about sensory experience and (2) to examine how the use of cooling flavors corresponds to report of broader, traditionally used flavor categories used. Additionally, since cooling additives have been shown to contribute to the palatability of nicotine in e-cigarettes [5, 6], we also examine the association of cooling flavor use to vaping frequency in the past 30 days and the use of nicotine e-liquids.

## Methods

### Participant and procedures

In Spring 2019, students from 6 Connecticut high schools (N = 4875) completed twenty-minute, anonymous, tablet-based surveys assessing tobacco product use. This survey was one in a series of longitudinal surveys assessing tobacco use among youth in a cohort of schools. Initial survey content was developed based on focus groups conducted with middle and high school youth and each wave of the survey is updated by content experts to adapt questions based on concepts identified in earlier surveys and introduce questions based on the changing marketplace for new and emerging tobacco products. Schools were selected from distinct District Reference Groups, which classify Connecticut school districts based on family income levels, parental education and occupation, and use of non-English language in the home. Survey administration has been described previously [16]. Parents were sent information letters and could decline their child’s participation. Students were informed participation was voluntary and anonymous. Surveys were conducted in-person on school grounds and survey completion was considered consent/assent. Students received a stylus for participating in the survey. The Yale School of Medicine Institutional Review Board and the participating high schools approved the study.

### Measures

#### Demographics

Participants reported on their sex at birth (male/female), age (13–19 years), and race/ethnicity. For all analyses, race/ethnicity was coded as Hispanic, non-Hispanic (NH) Black, NH White, and NH Other Race.

#### Use of cooling e-liquid flavors

Participants who indicated ≥1 day of e-cigarette use in the past 30 days (“Approximately how many days out of the past 30 days did you vape?”; n = 1326) were asked “In the past 30 days, did you vape flavors that produce a cooling sensation in your mouth or throat (freeze, ice, chill)?” Response options were: 1) Yes, 2) No, and 3) I don’t know. 1095 of participants who report past 30-day e-cigarette use (82.6%) participants indicated a response for this item. Those that responded “I don’t know” were not included in the current analyses (n = 179).

#### E-cigarette use characteristics

Participants reported on past 30-day use of five types of e-cigarette devices: cig-a-likes, vape-pens, JUUL, other pod systems, and Mods/APVs; all devices were accompanied by a product description and product images. Past-30 day nicotine use (dichotomized as yes/no) was determined by youth reporting if they had used nicotine in any of the devices the report past 30 day use of. Participants also reported past-30-day e-cigarette use frequency as the total number of days out of the past 30 days when they used any e-cigarette device. Additionally, participants reported on age at vaping onset and on the e-liquid flavor categories they had used in the past 30 days (i.e., tobacco, menthol, mint, fruit, candy, vanilla, coffee, spice, alcohol, other). The total number of flavor categories used in the past month was calculated by summing the flavor categories endorsed by individual.

#### Statistical analysis

The analytic sample included 916 survey respondents who reported past 30-day e-cigarette use and indicated yes or no to the question asking about cooling flavor use. We used chi-square analyses and independent sample t-tests to examine whether demographics, past 30-day nicotine e-liquid use, past 30-day vaping frequency, total number of flavor categories used and/or past 30-day e-liquid flavor category types use differed between those who reported vaping flavors that produce a cooling sensation (“cooling flavors”) and those who did not. Cohen’s D effect sizes were reported for continuous variables and Cramer’s V effect sizes were reported for categorical variables.

We then examined the association between e-cigarette use behaviors and use of cooling flavors when adjusting for additional covariates associated with e-cigarette use. Specifically, we conducted a binary logistic regression to examine if past 30-day nicotine e-liquid use and/or past 30-day frequency of e-cigarette use was associated with vaping cooling flavors (compared to not vaping cooling flavors). We included vaping age of onset in the model (which has been associated with heavier e-cigarette use [17]), total number of flavors used (to account for any differences between groups related to heavier flavor use overall), age, sex, race/ethnicity, and school as covariates. SPSS Version 26 was used to conduct all analyses. For all analyses, the threshold for statistical significance was *p* < .05.

## Results

### Descriptive findings

The analytic sample (n = 916) was 56.2% female and had a mean age of16 years (SD: 1.3, Range: 13–19). Race/ethnicity of the sample was 53.5% NH White, 27.3% Hispanic, 14.0% NH Black, and 5.1% NH Other Race. Approximately half of the sample (51.6%; n = 473) reported vaping cooling flavors. Bivariate associations are presented in Table 1. Those who vaped cooling flavors did not differ from those who did not vape cooling flavors by demographics. Youth who vaped cooling flavors reported greater past 30-day vaping frequency and higher rates of past 30-day use of nicotine e-liquids, than those who did not vape cooling flavors. Total number of flavor categories used in the past 30 days and past 30-day rates of use of flavor categories menthol, mint, fruit, vanilla, spice, alcohol, and tobacco were higher among those vaping cooling flavors (Table 1).

**Table 1:** Demographic and e-cigarette use of youth who do and do not vape cooling flavors.

	Vape Cooling Flavors (n = 473)	Do Not Vape Cooling Flavors (n = 443)	*p* value	Effect Size
Gender (% Female)	54.7%	58.0%	0.31	0.03
Age (M, SD)	16.23 (1.28)	16.26 (1.23)	0.74	0.02
Race			0.41	0.06
NH Black (%)	14.6%	13.6%		
Hispanic (%)	26.0%	28.6%		
NH Other (%)	4.2%	6.1%		
NH White (%)	55.2%	51.6%		
E-Cigarette Use				
**Vaping Age of Onset (M, SD)**	13.66 (1.92)	14.24 (1.75)	< .001	0.31
**Past 30-day Nicotine Use in E-cigarettes (%)**	91.70%	74.80%	< .001	0.23
**Past 30-day Frequency (Days) of E-Cigarette Use (M, SD)**	18.71 (11.18)	12.18 (11.08)	< .001	0.56
**Past 30-day, Total Number of Flavors Vaped (M, SD)**	2.21 (1.73)	1.39 (1.15)	< .001	0.53
Past Month E-Cigarette Flavor Categories				
**Tobacco (%)**	11.5%	5.0%	0.001	0.12
**Menthol (%)**	24.2%	9.0%	< .000	0.20
**Mint (%)**	81.4%	56.7%	< .001	0.27
**Fruit (%)**	59.6%	48.2%	0.001	0.11
Candy (%)	16.4%	13.9%	0.31	0.04
**Vanilla (%)**	11.6%	6.9%	0.03	0.08
Coffee (%)	7.6%	5.6%	0.27	0.04
**Spice (%)**	4.0%	1.1%	0.01	0.09
**Alcohol (%)**	6.0%	2.8%	0.03	0.08
Other (%)	2.2%	1.4%	0.38	0.03

Note: (M, SD) in the table is representative of mean and standard deviation, respectively. NH indicates “Non-Hispanic”. Bolding indicates significance at *p* < .05. Comparisons between means were calculated with two-tailed t-tests and comparisons between percentages were calculated using Chi-square analysis. Effect size reported as Cohen’s D presented for continuous variables and Cramer’s V for chi square analyses.

### Adjusted findings

After adjusting for covariates (Table 2), past 30-day nicotine e-liquid use (AOR = 1.95 [95%CI: 1.23, 3.09]) and greater vaping frequency (AOR = 1.03 [95% CI: 1.01, 1.04]) were associated with increased odds of reporting vaping cooling flavors. An indication of yes to past 30-day nicotine e-liquid use was associated with increase odds of vaping cooling flavors by 1.95. Given the scaling of vaping frequency, every one-day increase in past 30-day vaping frequency increased odds of vaping cooling flavors by 1.03. For instance, if someone reports 20 days of vaping in the past 30-days, they would have a cumulative increased odds of 20.6 of using cooling flavors (vs. not using cooling flavors). Additionally, significant covariates included earlier age of onset of e-cigarette use which was associated with increased odds of vaping cooling flavors (AOR = 0.91, [95% CI: 0.83, 0.99]) and the total number of flavor categories vaped in the past 30-days (AOR = 1.39 [95% 1.22, 1.59]) in which greater number of flavor categories reported was associated with increased odds of vaping cooling flavors. No other covariates (age, sex, race/ethnicity, school) were significantly associated with vaping cooling flavors.

**Table 2:** Adjusted odds ratios for adolescents examining association with cooling flavors.

Independent Variables	AOR	95% CI		*P* value
Age	1.01	0.89	1.15	0.87
Male^a^	0.98	0.72	1.32	0.87
NH Black^b^	1.41	0.89	2.23	0.14
Hispanic^b^	1.22	0.83	1.80	0.31
NH Other Race^b^	1.02	0.48	2.15	0.96
**Vaping Age of Onset**	**0.91**	**0.83**	**0.99**	**0.03**
**Total Flavors Vaped, Past 30-days**	**1.39**	**1.22**	**3.09**	**< .001**
**Nicotine Use in E-Cigarettes, Past 30-days**	**1.95**	**1.23**	**3.09**	**< .001**
**Vaping Frequency, Past 30-days**	**1.03**	**1.01**	**1.04**	**< .001**

Note: bolded numbers have a p < .05. School was added to the model as a covariate (not shown).

^a^vs female

^b^vs Non-Hispanic White; missing cases (n = 75; 8.2%).

## Discussion

This study adds to the literature by providing new information on the association of youth e-cigarette use and cooling sensory experience. We observed that a sizeable proportion of adolescent e-cigarette users (51.6%) reported use of e-liquids that produced cooling sensations. Further, past 30-day nicotine e-liquid use and more frequent vaping in the past 30 days were associated with a greater likelihood of vaping cooling flavors. The fact that nicotine e-liquid use and vaping frequency remained significant after accounting for age of onset and the total number of flavors used in the past 30 days suggests that the observed findings were not driven solely by duration of e-cigarette use or heavier use of flavors. The higher rates of nicotine e-liquid use and greater vaping frequency observed among those who reported vaping cooling flavors could be related to reductions in nicotine aversiveness and increases in nicotine appeal as shown by prior experimental studies using cooling (i.e. menthol-flavored) e-liquids [5, 6, 10]. Given that additives that produce cooling may not be indicated by e-liquid product labeling, future studies need to characterize the expected sensory effects of flavors and constituents that produce these effects.

With regard to differences in past-month use of individual e-liquid flavor categories, not surprisingly, those who reported vaping cooling flavors reported higher rates of vaping menthol and mint flavors. However, youth who reported vaping cooling flavors also reported vaping fruit, vanilla, spice, alcohol, and tobacco flavor categories at higher rates. We could not determine which specific flavors categories respondents used that produced cooling flavors from our data, but future research should investigate which broader flavor categories used by youth most commonly produce cooling sensations. Importantly, when examining how the use of nicotine e-liquids and vaping frequency related to the use of cooling flavors, we included number of flavor categories used as a covariate in the model to ensure that our findings were not solely a function of heavier flavor use among this group.

Notably, some youth who indicated that they did **not** use cooling flavors endorsed vaping menthol (9.0% of those reporting not using cooling flavors) and mint (56.7% of those reporting not using cooling flavors) flavor categories. This suggests that some youth may not experience a cooling sensation when vaping these flavors or that they do not associate mint or menthol flavors with the description “producing a cooling sensation.” An alternative explanation for this could also be due to recall of sensory experience. It may be cognitively easier for youth to retrospectively recall a flavor name (commonly indicated on the packaging materials and product itself) than the sensory experience of cooling. Although this question was asking about past 30-day experience, a relatively short time frame, this may be an important consideration in the perception of flavor. This evidence suggests that more work is needed to understand how youth characterize cooling flavors and how that may differ from their report of the flavors they use. The current study suggests that it may be important to characterize tobacco products by the sensation(s) produced in addition to the characterizing flavor.

Our results should be interpreted in light of study limitations. First, data are from Connecticut high school students and may not be representative of the general population. However, this survey is one in a programmatic series, and, historically, the resulting data have closely mirrored national data [18]. Second, we could not discern what specific flavor categories (e.g., menthol, fruit) produced a cooling sensation since youth reported using multiple flavor categories. Future work should: determine 1) what flavors are perceived as producing a cooling sensation, 2) how many youth who report using cooling flavors use flavors that are not characterized as mentholated, and 3) if flavors that produce cooling but are not labeled as mentholated contain menthol or menthol-like additives and/or produce cooling effects in experimental studies. Finally, the wording of our question assessing cooling flavors could have impacted the results (“In the past 30 days, did you vape flavors that produce a cooling sensation in your mouth or throat (freeze, ice, chill)?”). These terms are commonly used to describe flavors with cooling components that may not be traditionally characterized as menthol or mint (e.g. Watermelon Ice). The addition of the example terms could have resulted in students endorsing “no” if they did not vape flavors with these specific descriptors. In the future, the experience of cooling should be assessed distinctly from flavor names, descriptors, or labels.

In conclusion, this study is the first to characterize e-cigarette use behavior among youth in the context of the sensory experience produced by a flavor (i.e., cooling) rather than the flavor category used (e.g. menthol, fruit). This method is more inclusive of flavors that may produce a cooling sensation but are not characterized as mentholated. As regulatory agencies, such as the FDA, move toward regulating menthol flavors in tobacco products [15], it is important to consider that flavor regulations should not focus only on particular flavor additives (e.g. menthol), but consider all additives that not only taste/smell like menthol, but create similar sensory experiences. In regard to e-cigarette regulation, identifying and restricting compounds that produce a particular sensory experience (i.e. cooling) in order to prevent companies from replacing menthol with other constituents that produce the cooling sensory experience and restricting youth access to these particular constituents may be potential mechanisms for reducing e-cigarette use among youth. The current study demonstrates that the sensory experience of cooling is associated with more frequent vaping and greater use of nicotine e-liquids. When considering the impact of flavor use on youth, we should examine how sensory experience may influence e-cigarette use behaviors and other product use in addition to the impact of particular additives on these behaviors.

## Supporting information

S1 Survey Question text and response options for the variables included in this study. (DOCX)

S1 Data Data file containing all the central study variables. (XLSX)
