# Supplementary material for: Cooling e-cigarette flavors and the association with e-cigarette use among a sample of high school students
Source: PLoS One. 2021 Sep 1;16(9):e0256844. doi: 10.1371/journal.pone.0256844 (PMC8409641; doi:10.1371/journal.pone.0256844)
Supplement: S1 Survey — (DOCX) [file pone.0256844.s001.docx]

How old are you?

- 13 (13)
- 14 (14)
- 15 (15)
- 16 (16)
- 17 (17)
- 18 (18)
- 19 (19)

At birth, what was your sex?

- Female (0)
- Male (1)

Are you Hispanic, Latino, or of Spanish origin?

- No (0)
- Yes (1)

How would you describe your RACE? (SELECT ALL THAT APPLY)

- White (1)
- Black or African American (2)
- Asian (3)
- American Indian or Alaska Native (4)
- Native Hawaiian or Pacific Islander (5)
- Middle Eastern (6)
- Other (please specify) (7)

How old were you when you first VAPED, even just 1 or 2 puffs?

- 8 or younger (8)
- 9 (9)
- 10 (10)
- 11 (11)
- 12 (12)
- 13 (13)
- 14 (14)
- 15 (15)
- 16 (16)
- 17 (17)
- 18 (18)
- 19 (19)

Approximately how many days out of the PAST 30 DAYS did you VAPE? 
When answering, please think about the TOTAL NUMBER OF DAYS that you vaped using ANY TYPE OF DEVICE.

- 0 (0)
- 1 (1)
- 2 (2)
- 3 (3)
- 4 (4)
- 5 (5)
- 6 (6)
- 7 (7)
- 8 (8)
- 9 (9)
- 10 (10)
- 11 (11)
- 12 (12)
- 13 (13)
- 14 (14)
- 15 (15)
- 16 (16)
- 17 (17)
- 18 (18)
- 19 (19)
- 20 (20)
- 21 (21)
- 22 (22)
- 23 (23)
- 24 (24)
- 25 (25)
- 26 (26)
- 27 (27)
- 28 (28)
- 29 (29)
- 30 (everyday) (30)

In the PAST 30 DAYS, which flavors did you VAPE?
 (SELECT ALL THAT APPLY)

- Tobacco (1)
- Menthol (2)
- Mint (spearmint, wintergreen, peppermint) (3)
- Fruit (cherry, blueberry, strawberry, coconut) (4)
- Candy or dessert (chocolate, Jolly Rancher) (5)
- Vanilla (6)
- Coffee (espresso, latte, cappuccino) (7)
- Spice (clove, cinnamon, nutmeg) (8)
- Alcohol (pina colada, strawberry daiquiri, bourbon, rum, brandy) (9)
- Other (please specify) (10) ________________________________________________
- ⊗I have not used any of these e-liquid flavors in the past 30 days. (12)

IN THE PAST 30 DAYS, did you VAPE flavors that produce a cooling sensation in your mouth or throat (freeze, ice, chill)?

- No (0)
- Yes (1)
- I don't know (2)
